# Mortality in children aged 0–14 years born to immigrant parents in Sweden: a total-population cohort study

**DOI:** 10.1016/j.lanepe.2026.101666

**Published:** 2026-04-02

**Authors:** Anastasia Lam, Ben Wilson, Matthew Wallace

**Affiliations:** a Department of Social Sciences, Humboldt-Universität zu Berlin, Berlin, Germany; b Einstein Center Population Diversity (ECPD), Berlin, Germany; c Department of Sociology, University of Stockholm, Stockholm, Sweden; d Department of Methodology, London School of Economics, London, UK; e Centre for Research on Inclusive Society, School of Health and Society, University of Salford, Salford, UK

**Keywords:** Child mortality, Second generation, Immigrants, Health inequalities, Sweden, Social determinants of health, Population-based cohort study

## Abstract

**Background:**

Children of immigrants in Europe have higher infant and early-adult mortality, compared with non-immigrants or their children. We aim to establish whether they have an increased risk of child mortality, focussing on the case of Sweden.

**Methods:**

We use Cox proportional hazards regression models to estimate hazard ratios (HRs) using Swedish total-population administrative data from 1990 to 2019. We estimate all-cause mortality for children aged 0–14, overall and separately for ages 0, 1–4, and 5–14. We do this for Swedish-born children of immigrants with either one or two foreign-born parents, across a wide range of origins. HRs are reported with reference to child mortality for Swedish-born children with two Swedish-born parents.

**Findings:**

We find persistently higher mortality for children of immigrants with two parents born in the Middle East & Northern Africa (HR ages 0–14 = 1.22 for mother's country of birth (95% CI: 1.13–1.33)). Inequalities exist at all childhood ages but we find scant evidence of higher mortality risks in the same origin groups when only one parent is foreign-born.

**Interpretation:**

A higher risk of childhood death in these groups is the result of specific factors that interact uniquely across ages and origins. While mortality at these ages is low in absolute terms, each death is a tragedy that represents decades of life lost and has far-reaching social consequences.

**Funding:**

10.13039/501100000781European Research Council, United Kingdom Research and Innovation (through the Horizon Europe Guarantee programme), 10.13039/501100004359Swedish Research Council, Swedish Research Council for Health, Working Life and Welfare, and the Scottish Graduate School of Social Science–Scottish Funding Council.

**Research in context**

**Evidence before this study**

Our searches for peer-reviewed publications were conducted in *Scopus*, *PubMed* and *Web of Science* with no restriction on publication dates or language(s). Strings included variations on terms associated with ‘migrant(s)’ (e.g. immigrant, foreign-born, nativity, ethnicity), ‘mortality’ (e.g. survival, death, longevity, life expectancy), ‘morbidity’ (e.g. health, disease, illness, medical conditions), and/or the term ‘paradox’. We did not impose strict selection criteria. The searches uncovered a 2023 review of mortality among the children of immigrants in Europe that reported elevated infant (stillbirth, perinatal, neonatal and infant mortality) and adult mortality in this group, notably if parent(s) were born outside of Europe in the Middle East and Sub-Saharan Africa. Social disadvantage played an important role in attenuating these increased risks. By the review's own admission, studies focusing on mortality after infancy and before adulthood were rare. Nearly all empirical studies were limited to studies in low and middle-income contexts. Nevertheless, a 1999 study from the Netherlands and a 2011 study from Denmark both reported elevated risks of child mortality (for children aged under-15, and aged under-5, respectively) among the children of Turkish and Moroccan mothers in the Netherlands, and among Turkish, Pakistani, Somali and Iraqi mothers residing in Denmark.

**Added value of this study**

The present study adds to the scant evidence on disparities in child mortality among the children of immigrants in high-income countries. We contribute detailed, reliable, and robust estimates derived from total population administrative data in Sweden at ages 0–14, age 0, ages 1–4 and ages 5–14 over a long time period (1990–2019). We report estimates for children of immigrants with two foreign-born parents (the G2.0) *and* one foreign-born parent (the G2.5), by maternal *and* paternal country of birth, and both before *and* after adjusting a set of relevant confounders. We find differences by generation, observing evidence of increased mortality risks for many children of immigrants with *two* foreign-born parents, but largely a lack of evidence for those with *one* foreign-born parent. However, the size and direction of mortality differentials are consistent, irrespective of whether we use the country of birth of the mother or the country of birth of the father. Finally, we find considerable heterogeneity by parental origin, such that children of immigrants born in the Middle East and Northern Africa have persistently higher mortality risks at all childhood ages, even after adjusting for a range of observed confounders.

**Implications of all the available evidence**

A clear picture is now developing in high-income countries of higher childhood mortality risks for children of immigrants with a non-Western background. Child mortality is almost entirely preventable through effective intervention. Even if mortality in absolute terms is low in high-income countries, every child's death is a tragedy for the family, friends, caregivers and communitities in which children live. The presence of systematic disparities in child mortality relative to children of non-immigrants suggests that the children of immigrants are being failed by social policies. Renewed efforts should be made to tackle both upstream and downstream determinants of child health and mortality among the children of immigrants—and their parents—in order to generate greater equity in outcomes and opportuities.

## Introduction

Children of immigrants are one of the fastest-growing and most diverse young populations in high-income countries around the world today.^1^ This owes to the rising volume, visibility, and diversification of migration to high-income countries over the past half a century or so.^2^ Immigrants’ children represent an integral part of the national tapestry of many high-income countries, and their health plays a key role in determining the overall health of the child population.^1^ A recent review article shows consistent evidence of higher mortality risks across Europe for children of immigrants during infancy (including *stillbirth*, *perinatal*, *neonatal*, and *infant mortality*) and in early to mid-adulthood.^3^ The risk of mortality was highest among children with parents born outside Europe, notably the Middle East and Sub-Saharan Africa.^3^ Potential explanations include structural racism and discrimination, issues with access to healthcare systems and the suitability of those systems, failures of national and local immigrant integration processes, and inequalities in social determinants of health like education, housing, labour market outcomes, and socioeconomic status.^3^

In contrast, there has been scant research on mortality among the children of immigrants after infancy and prior to adulthood (i.e., ages 1–14-years-old) in high-income countries.^3^ This represents a salient omission given that these are the ages in which we find some of the largest shares of children of immigrants. In the EU in 2023, for example, children of immigrants accounted for 28% of the resident population aged less than 5-years-old, 30% of the resident population aged 5–9, and 27% of the resident population aged 10–14.^4^ This compares with less than 7% of the total resident population aged 15–74.^3^ While it holds true that child mortality is low in high-income countries (HICs; certainly compared to low-income countries, where reducing child mortality represents a global public health priority), substantial variations in child mortality persist within and across HICs.^5^ National systematic reviews of child mortality in high-income countries deem a sizeable proportion of child deaths to be entirely preventable, avoidable, and reflective of relative poverty, inequality, and acute failures within national healthcare systems.^6^

If there *is* variation in the risk of child mortality according to the migration background of parents in HICs, it becomes important to generate evidence that identifies the groups most at risk and in need of support from policies. Equally, it is vital to address the gap in evidence about mortality risks that exists for the period between infancy and adulthood among children of immigrants in HICs. Such evidence contributes to a more complete picture of the mortality risks that children of immigrants face in HICs and has the potential to lay the foundation for future scholarship on health inequalities during this critical period of the life course.

In order to address this evidence gap, we carry out a cohort study in Sweden, which is an ideal study context given its high-quality longitudinal data for the whole population, and for its long history of receiving substantial numbers of immigrants. In 2019, children born to at least one immigrant parent comprised 29% of the total Swedish population aged 0–14, up from 16% in 1990.^7^

Our study aims to establish whether Swedish-born children with any foreign-born parent(s) have an increased risk of under-15 mortality between 1990 and 2019, as compared with those who have two Swedish-born parents. In doing so, we also set out to examine variation by parental country of birth, for mothers and fathers—including for children with one foreign-born and one Swedish-born parent—as well as the role of a range of socioeconomic factors in moderating the patterns that we observe. Based on prior research,^3^^,^^7^^,^^8^ we expect that there will be a difference between those who have two foreign-born parents (G2) and those who have one foreign-born and one native-born parent (G2.5), since the latter have been shown to experience lower rates of mortality in adulthood. However, we do not expect material differences between children who have mothers from a specific origin as compared with those who have fathers from the same origin, although such differences may be hypothesized given known differences between the social and biological role of mothers as compared with fathers, in particular at younger ages (e.g. infancy).^9^ With respect to socioeconomic factors, we expect that social disadvantage will be associated with higher child mortality, in line with prior work on the social determinants of health.^10^^,^^11^

## Methods

### Data

We used collections of high-quality administrative microdata that include detailed information for the entire registered population of Sweden, including linkages between multiple generations of immigrants and their children.^12^, ^13^, ^14^ Through the availability of individual and family identifiers, children are linked to their parents to identify their parent's country of birth, refugee status and socioeconomic status (at the child's year of birth), as well as maternal age at birth.

Individuals were eligible to enter the study population if they were born in Sweden between 1990 and 2019, and leave the population if they emigrated or died before age 15-years-old (n = 3,247,164). A flowchart describing the process by which we obtained our final study population is provided in Supplementary Figure S1. Given our focus on comparisons within and between the nativity of mothers and fathers, eligibility was also requisite on having two biological parents, such that we excluded children who were adopted or had no biological father in the registers (n = 48,279). Of all eligible cases (n = 3,198,406), we then dropped approximately 3% due to missing data, the majority of which (2.6%) were missing mother's education, with the rest missing parental marital status, residence status, or father's country of birth.

### Study variables

The main outcome was all-cause mortality before age 15, derived from both the death register and medical birth register. We also analysed mortality separately for infants (<1 year old), children aged 1–4 years old, and children aged 5–14 years old to test whether differences between these age groups might help to explain the overall differences in mortality.

Exposure was parental country of birth (derived from the population registers), categorised into: Sweden, Finland, Other Nordic, Other Western, Eastern Europe, Middle East & Northern Africa, Sub-Saharan Africa, Central & South America, and Asia & Oceania. Second generation (G2.0) children were defined as having two parents born outside Sweden. The G2.5 were defined as children with one Swedish-born parent and one foreign-born parent.

Adjustment variables included sex, birth order, birth year, maternal age, mother's education, residence type, marital status, and household disposable income. Sex was defined as ‘male’ or ‘female’ and derived from the population registers. Maternal age was the age of the mother at the birth of the child and was derived from the birth years of mother and child from the population registers. Mother's education was the highest level of attained education in the child's birth year, categorised as ‘low’, ‘medium’, and ‘high’, and was used because it is less likely to be missing than father's education. Residence type was time-varying and dichotomised as ‘urban’ or ‘rural’, with urban defined as Stockholm County, Skåne County, and Västra Götaland County. Marital status was time-varying and categorised as ‘married or partnered’, ‘cohabiting’, or ‘single’. Household disposable income was time-varying, included earnings and social allowances, and was categorised into quintiles. Mother's education and disposable income were both derived from the Longitudinal Integrated Database for Health Insurance and Labour Market Studies (LISA).

### Statistical analysis

To assess the association between parental country of birth and child mortality, we used Cox proportional hazards regression models to estimate hazard ratios (HRs) with 95% confidence intervals (CIs) and robust standard errors clustered using mother's ID (although all models use whole-population data, so these intervals do not represent uncertainty with respect to population inference). Individuals entered the risk set at their birth between 1990 and 2019 and exited the risk set if they died, emigrated, became older than 14-years-old, or were alive and resident in Sweden on 31 December 2019. We interacted generation (G2.0 vs. G2.5) and either mother's or father's country of birth, since for the G2.0, parents can come from different origin countries. Models were fitted separately for mothers' and fathers' origins, and the reference group was Swedish-born children with two Swedish-born parents. All models were adjusted for sex, birth order, birth year, maternal age, residence, and marital status. Subsequent models then included mother's education (Models 1b and 2b) and household disposable income quintile (Models 1c and 2c). We assessed proportional hazards assumptions using Schoenfeld residual-based tests. To account for heterogeneity in mortality risk over age, all models allow for age-group specific baseline hazards and include time-varying residence type, parental marital status, and household disposable income. Age-stratified analyses were also performed. All analyses were conducted in R version 4.4.2.

### Sensitivity analysis

We conducted additional analysis specifying parental refugee status as the exposure (instead of parental country of birth). Here, refugee status was defined as whether either parent has ever been a refugee (based on their residence permit history in the population registers). We also ran models using father's education instead of mother's, both mother's and father's education, and included missing categories for mother's education and father's country of birth instead of excluding them. We stratified by 10-year periods (1990–1999, 2000–2009 and 2010–2019) to assess whether there might be any time trends in mortality. Additionally, we stratified by generation (G2.0 and G2.5) to examine how the relationship between exposure and outcome may vary across covariates.

### Ethics approval

These data were accessed and analysed under ethical approval from the Swedish Ethical Review Authority (reference: 2021-03533). Access was provided by Statistics Sweden after pseudo-anonymizing the data for research purposes.

### Role of the funding source

The funders had no role in study design, in the collection, analysis, and interpretation of data, in the writing of this article, nor in the decision to submit this paper for publication.

## Results

Table 1 provides characteristics of our sample (N = 3,100,245), of which 14% (n = 418,878) were G2.0 and 13% (n = 388,966) were G2.5. Mothers of G2.0 children tended to be disadvantaged in terms of their education and disposable income compared with G2.5 children and Swedish-born children who have two Swedish-born parents. Among the G2.0, a large share had mothers (36%) and fathers (38%) from the Middle East & Northern Africa. Conversely, among the G2.5, apart from Sweden, the largest groups were those with mothers from Asia & Oceania (12%) or fathers from Other Western origins (17%). Moreover, while 30% of G2.0 mothers and 44% of G2.0 fathers have ever held a refugee permit in Sweden, the equivalent percentages for the G2.5 are only 4% and 7%, respectively. Crude unadjusted mortality rates in infancy were highest among the G2.0 (650.15 per 100,000 person-years), followed by the G2.5 (530.13) then the Swedish-born to two Swedish-born parents (523.22). The G2.0 also had the highest rates at ages 1–4 (G2.0: 22.59, Swedish-born to two Swedish-born parents: 16.80, G2.5: 15.99), and ages 5–14 (G2.0: 9.88, G2.5: 8.44, Swedish-born to two Swedish-born parents: 7.91). Prior to any adjustment, this already suggests the presence of some material disparities.

**Table 1:** Sample characteristics.

	Swedish-born with Swedish-born parents (N = 2,292,401)	G2.0 (N = 418,878)	G2.5 (N = 388,966)
**Sex**			
Male	1,178,361 (51.40%)	215,154 (51.36%)	199,574 (51.31%)
Female	1,114,040 (48.60%)	203,724 (48.64%)	189,392 (48.69%)
**Birth order**			
Mean (SD)	1.82 (0.94)	2.18 (1.31)	1.81 (0.98)
**Maternal age**			
Mean (SD)	29.78 (4.99)	29.73 (5.47)	30.38 (5.38)
**Family type (at birth year of child)**			
Married or partnered	934,829 (40.78%)	307,172 (73.33%)	195,370 (50.23%)
Cohabiting	1,190,341 (51.93%)	52,229 (12.47%)	140,221 (36.05%)
Single	167,231 (7.30%)	59,477 (14.20%)	53,375 (13.72%)
**Residence type (at birth year of child)**			
Urban	1,089,648 (47.53%)	270,329 (64.54%)	240,482 (61.83%)
Rural	1,202,753 (52.47%)	148,549 (35.46%)	148,484 (38.17%)
**Mother's education (at birth year of child)**			
Low	208,538 (9.10%)	126,875 (30.29%)	53,562 (13.77%)
Middle	1,085,624 (47.36%)	147,018 (35.10%)	158,594 (40.77%)
High	998,239 (43.55%)	144,985 (34.61%)	176,810 (45.46%)
**Disposable income quintiles (at birth year of child)**			
Lowest	341,066 (14.88%)	236,540 (56.47%)	120,957 (31.10%)
Lower	723,530 (31.56%)	110,580 (26.40%)	108,928 (28.01%)
Middle	541,720 (23.63%)	36,878 (8.80%)	66,193 (17.02%)
Higher	367,857 (16.05%)	20,190 (4.82%)	47,595 (12.24%)
Highest	318,228 (13.88%)	14,690 (3.51%)	45,293 (11.64%)
**Mother's country of birth**			
Sweden	2,292,401 (100.00%)	–	212,012 (54.51%)
Central & South America	–	16,765 (4.00%)	17,644 (4.54%)
Eastern Europe	–	32,459 (7.75%)	18,951 (4.87%)
Other Western	–	76,179 (18.19%)	34,945 (8.98%)
Other Nordic	–	8038 (1.92%)	17,840 (4.59%)
Sub-Saharan Africa	–	58,275 (13.91%)	6344 (1.63%)
Finland	–	12,218 (2.92%)	25,637 (6.59%)
Asia & Oceania	–	63,939 (15.26%)	46,822 (12.04%)
Middle East & Northern Africa	–	151,005 (36.05%)	8771 (2.26%)
**Father's country of birth**			
Sweden	2,292,401 (100.00%)	–	176,954 (45.59%)
Central & South America	–	16,349 (3.90%)	22,044 (5.67%)
Eastern Europe	–	25,697 (6.14%)	10,649 (2.74%)
Other Western	–	77,947 (18.61%)	66,056 (16.98%)
Other Nordic	–	8432 (2.01%)	20,904 (5.37%)
Asia & Oceania	–	62,483 (14.92%)	23,999 (6.17%)
Sub-Saharan Africa	–	60,487 (14.44%)	12,237 (3.15%)
Finland	–	10,197 (2.43%)	23,264 (5.98%)
Middle East & Northern Africa	–	157,286 (37.55%)	32,859 (8.45%)
**Mother has ever had a refugee permit in Sweden**			
No	2,291,883 (99.98%)	294,394 (70.28%)	375,069 (96.43%)
Yes	518 (0.02%)	124,484 (29.72%)	13,897 (3.57%)
**Father has ever had a refugee permit in Sweden**			
No	2,291,703 (99.97%)	235,061 (56.12%)	361,466 (92.93%)
Yes	698 (0.03%)	183,817 (43.88%)	27,500 (7.07%)
**Death in infancy**			
No	2,285,265 (99.73%)	413,543 (99.68%)	386,849 (99.73%)
Yes	6208 (0.27%)	1350 (0.32%)	1043 (0.27%)
**Death from age 1 to 4**			
No	2,213,572 (99.95%)	388,714 (99.94%)	369,196 (99.96%)
Yes	1077 (0.05%)	243 (0.06%)	168 (0.04%)
**Death from age 5 to 14**			
No	1,997,347 (99.94%)	307,830 (99.95%)	320,714 (99.94%)
Yes	1403 (0.06%)	227 (0.05%)	223 (0.06%)
**Total deaths ages 0**–**14**			
	8688 (0.38%)	1820 (0.43%)	1434 (0.36%)
**Mortality rate (per 100,000 person-years)**			
Infant	523.22	650.15	530.13
Ages 1–4	16.80	22.59	15.99
Ages 5–14	7.91	9.88	8.44

Supplementary Figure S2 shows results from a minimally-adjusted model. This demonstrates that prior to controlling for regional, family, and socioeconomic variables, G2.0 children with parents from Asia & Oceania, Sub-Saharan Africa, and the Middle East & Northern Africa have higher mortality risks compared to Swedish-born children with two Swedish-born parents. Table 2 shows the HRs and 95% CIs for the adjusted models, by mother's and father's origin, so that we can see the impact of controlling for potential confounders. The HRs for child mortality by generation and parental origin are substantially attenuated compared with the minimally adjusted models, indicating that regional, family, and socioeconomic variables explained much of the elevated mortality risk, particularly for children with parents from Asia & Oceania and Sub-Saharan Africa. Nevertheless, G2.0 children with parents born in the Middle East & Northern Africa continued to exhibit higher hazards of mortality compared with Swedish-born children who have two Swedish-born parents.

**Table 2:** Hazard ratios and 95% confidence intervals of under-15 mortality among the children of immigrants according to generational status and the migration background of their parents, 1990–2019.

	Mother's country of birth			Father's country of birth		
	Model 1a	Model 1b	Model 1c	Model 2a	Model 2b	Model 2c
**G2.0 (Both parents born abroad)**						
Two parents born in Sweden (Reference)	1.00	1.00	1.00	1.00	1.00	1.00
Central & South America	0.71 (0.55–0.92)	0.68 (0.53–0.89)	0.67 (0.52–0.87)	0.71 (0.54–0.92)	0.69 (0.53–0.89)	0.67 (0.52–0.88)
Eastern Europe	0.75 (0.61–0.92)	0.75 (0.61–0.92)	0.73 (0.59–0.90)	0.79 (0.63–1.00)	0.79 (0.63–1.00)	0.77 (0.61–0.97)
Other Western	0.94 (0.83–1.07)	0.91 (0.81–1.03)	0.89 (0.78–1.01)	0.93 (0.82–1.05)	0.90 (0.79–1.02)	0.88 (0.77–0.99)
Other Nordic	1.00 (0.70–1.43)	0.99 (0.69–1.42)	0.96 (0.67–1.38)	0.99 (0.69–1.42)	0.98 (0.69–1.41)	0.95 (0.66–1.36)
Asia and Oceania	1.07 (0.94–1.21)	1.03 (0.91–1.17)	0.99 (0.88–1.13)	1.05 (0.92–1.19)	1.01 (0.89–1.15)	0.97 (0.86–1.11)
Sub-Saharan Africa	1.09 (0.96–1.23)	1.02 (0.90–1.15)	0.99 (0.87–1.12)	1.07 (0.95–1.21)	1.00 (0.89–1.13)	0.97 (0.86–1.10)
Finland	1.05 (0.83–1.33)	1.03 (0.81–1.30)	1.02 (0.80–1.28)	1.13 (0.88–1.45)	1.09 (0.85–1.40)	1.08 (0.84–1.39)
Middle East & Northern Africa	1.36 (1.26–1.46)	1.28 (1.19–1.39)	1.22 (1.13–1.33)	1.34 (1.24–1.44)	1.27 (1.17–1.37)	1.21 (1.12–1.31)
**G2.5 (One parent born abroad)**						
Two parents born in Sweden (Reference)	1.00	1.00	1.00	1.00	1.00	1.00
Sweden^a^	0.83 (0.77–0.90)	0.82 (0.76–0.89)	0.81 (0.75–0.87)	1.04 (0.96–1.12)	1.03 (0.95–1.11)	1.02 (0.94–1.10)
Central & South America	1.01 (0.79–1.29)	1.00 (0.78–1.27)	0.99 (0.77–1.26)	0.62 (0.48–0.81)	0.62 (0.47–0.80)	0.61 (0.47–0.79)
Eastern Europe	1.07 (0.85–1.34)	1.08 (0.86–1.35)	1.07 (0.85–1.34)	0.76 (0.53–1.08)	0.75 (0.53–1.07)	0.74 (0.52–1.06)
Other Western	0.98 (0.82–1.17)	0.99 (0.83–1.18)	0.98 (0.82–1.17)	0.87 (0.76–0.99)	0.86 (0.76–0.98)	0.85 (0.74–0.97)
Other Nordic	1.16 (0.93–1.43)	1.15 (0.93–1.42)	1.14 (0.92–1.41)	1.09 (0.89–1.34)	1.08 (0.89–1.33)	1.07 (0.87–1.31)
Asia and Oceania	1.04 (0.89–1.21)	1.01 (0.86–1.18)	1.00 (0.86–1.17)	0.81 (0.65–1.02)	0.81 (0.64–1.02)	0.80 (0.63–1.00)
Sub-Saharan Africa	0.65 (0.40–1.07)	0.64 (0.39–1.04)	0.63 (0.39–1.03)	0.51 (0.35–0.74)	0.51 (0.35–0.73)	0.49 (0.34–0.71)
Finland	1.18 (1.00–1.39)	1.17 (0.99–1.38)	1.17 (0.99–1.37)	0.87 (0.71–1.06)	0.85 (0.70–1.04)	0.85 (0.70–1.03)
Middle East & Northern Africa	0.66 (0.43–1.03)	0.64 (0.41–1.00)	0.63 (0.41–0.98)	0.87 (0.72–1.04)	0.84 (0.70–1.00)	0.82 (0.68–0.98)
**Sex**						
Male (Reference)	1.00	1.00	1.00	1.00	1.00	1.00
Female	0.82 (0.79–0.85)	0.82 (0.79–0.85)	0.82 (0.79–0.85)	0.82 (0.79–0.85)	0.82 (0.79–0.85)	0.82 (0.79–0.85)
**Birth order**						
	1.02 (1.00–1.03)	0.99 (0.97–1.01)	0.99 (0.98–1.01)	1.02 (1.00–1.04)	0.99 (0.97–1.01)	0.99 (0.98–1.01)
**Birth year**						
	0.97 (0.97–0.97)	0.97 (0.97–0.97)	0.97 (0.97–0.97)	0.97 (0.97–0.97)	0.97 (0.97–0.97)	0.97 (0.97–0.97)
**Maternal age**						
	1.01 (1.00–1.01)	1.01 (1.01–1.02)	1.01 (1.01–1.02)	1.00 (1.00–1.01)	1.01 (1.01–1.02)	1.01 (1.01–1.02)
**Residence**						
Urban (Reference)	1.00	1.00	1.00	1.00	1.00	1.00
Rural	1.26 (1.21–1.31)	1.25 (1.21–1.30)	1.26 (1.21–1.30)	1.26 (1.21–1.31)	1.25 (1.21–1.30)	1.26 (1.21–1.30)
**Marital status**						
Married or partnered (Reference)	1.00	1.00	1.00	1.00	1.00	1.00
Cohabiting	0.48 (0.46–0.50)	0.47 (0.44–0.49)	0.47 (0.45–0.49)	0.48 (0.46–0.50)	0.47 (0.44–0.49)	0.47 (0.45–0.49)
Single	2.26 (2.16–2.37)	2.16 (2.06–2.26)	2.00 (1.89–2.12)	2.27 (2.17–2.37)	2.16 (2.06–2.26)	2.01 (1.90–2.12)
**Mother's education**						
Low (Reference)	–	1.00	1.00	–	1.00	1.00
Medium	–	0.88 (0.84–0.93)	0.89 (0.85–0.94)	–	0.88 (0.84–0.93)	0.89 (0.85–0.94)
High	–	0.76 (0.72–0.81)	0.77 (0.72–0.82)	–	0.76 (0.72–0.81)	0.77 (0.72–0.82)
**Household disposable income quintile**						
Lowest (Reference)	–	–	1.00	–	–	1.00
Lower	–	–	0.90 (0.85–0.95)	–	–	0.90 (0.85–0.95)
Middle	–	–	0.84 (0.78–0.89)	–	–	0.83 (0.78–0.89)
Higher	–	–	0.88 (0.82–0.94)	–	–	0.88 (0.82–0.94)
Highest	–	–	0.96 (0.89–1.03)	–	–	0.95 (0.89–1.03)

a If one parent was born in Sweden, the other parent was born abroad.

**Fig. 1:**
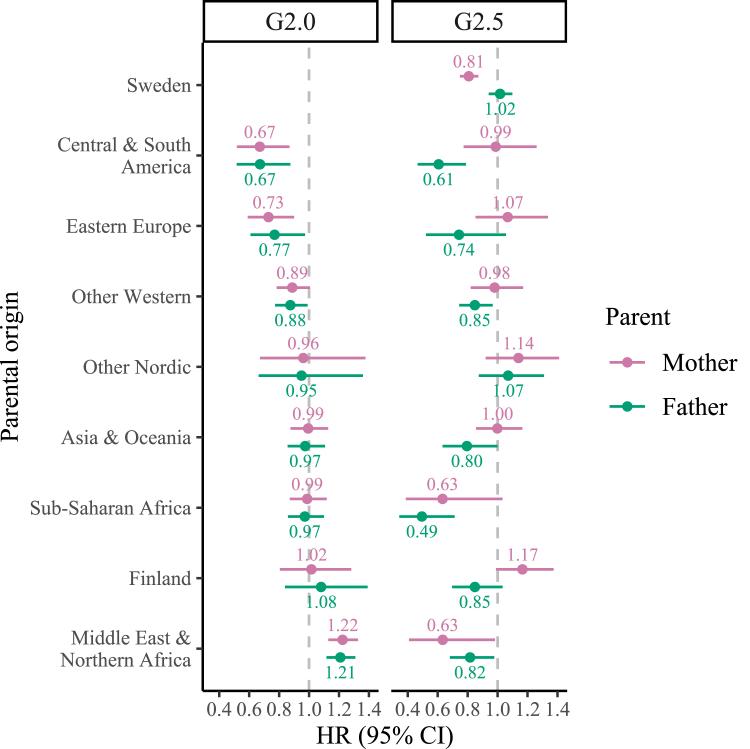
Fully-adjusted hazard ratios of under-15 mortality among the children of immigrants according to generational status and the migration background of the parents, 1990–2019. *Note.* Models are adjusted for sex, birth year, birth order, maternal age, mother's education, residence, marital status, and household disposable income. The reference group are children born in Sweden to two parents born in Sweden.

Fig. 1 displays HRs and 95% CIs of under-15 mortality from the fully-adjusted Cox model for G2.0 and G2.5 children, by parental origins, with reference to Swedish-born children with two Swedish-born parents. In the G2.0 panel (based on mother's country of birth), only one group stands out as having a higher hazard rate of child mortality compared with the reference group: Middle East & Northern Africa (HR: 1.22, 95% CI: 1.13–1.33). There is little difference in the size and direction of the HRs when using mother's or father's country of birth. Switching to the G2.5 panel, the patterns differ radically. *None* of the groups have materially higher HRs in the G2.5 panel. Indeed, only children with mothers born in Finland (HR: 1.17, 95% CI: 0.99–1.37) or Other Nordic countries (HR: 1.14, 95% CI: 0.92–1.41) have a similarly high HR as the G2.0 children with parents from the Middle East & Northern Africa.

**Fig. 2:**
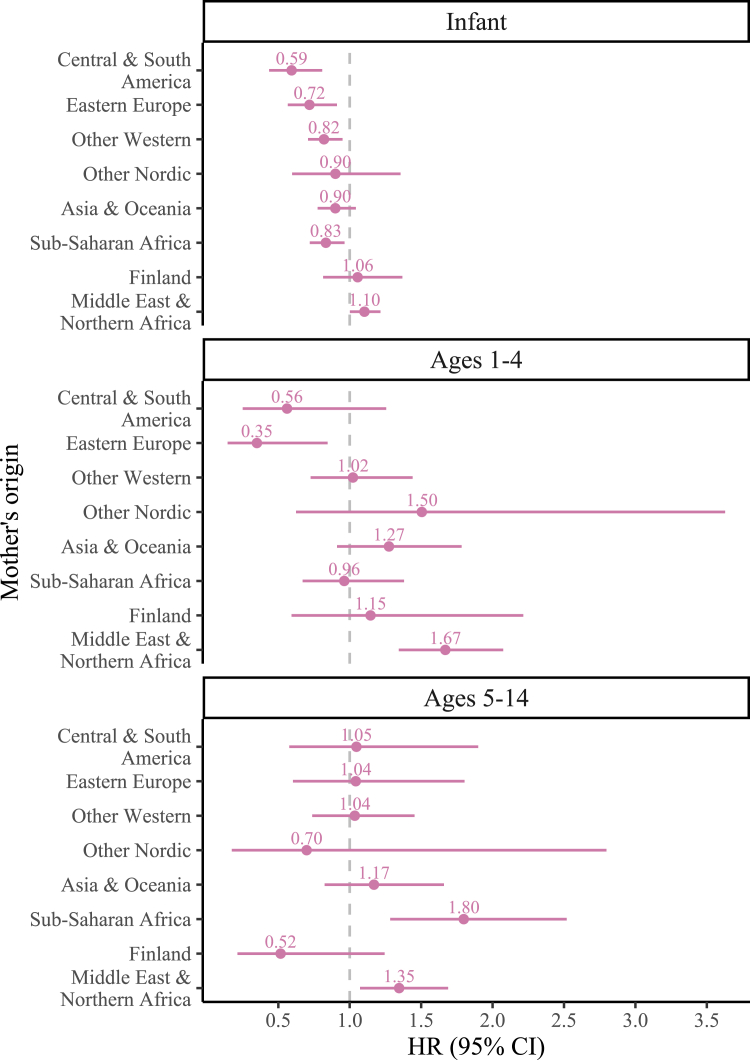
Fully-adjusted hazard ratios of infant mortality, 1–4 years old mortality and, 5–14 years old mortality among second generation children of immigrants according to the migration background of the mother, 1990–2019. *Note.* Models are adjusted for sex, birth year, birth order, maternal age, mother's education, residence, marital status, and household disposable income. The reference group are children born in Sweden to two parents born in Sweden.

Fig. 2 displays estimates for G2.0 mortality from the fully-adjusted model when disaggregated by child's age and mother's origin. Exact HRs and 95% CIs can be seen in Supplementary Table S1. G2.0 children of mothers born in the Middle East & Northern Africa continued to have a higher risk of mortality across the three age groups, but especially at ages 1–4 (HR: 1.67, 95% CI: 1.34–2.07). Additionally, at ages 5–14, G2.0 children with mothers from Sub-Sarahan Africa have the highest HR of mortality (HR: 1.80, 95% CI: 1.28–2.52). G2.0 children with mothers from Asia & Oceania also appear at higher risk at ages 1–4 (HR: 1.27, 95% CI: 0.91–1.78).

### Sensitivity analyses

Supplementary analysis using parental refugee status showed that in the minimally adjusted models, the G2.0 had a higher hazard of mortality, regardless of whether or not at least one parent ever had refugee status, compared with Swedish-born children with two Swedish-born parents (Supplementary Table S2). However, in the fully-adjusted models, children with refugee parents no longer had a higher hazard of mortality. Models using father's education instead of mother's and using both parents' education had results comparable with our main analysis (Supplementary Tables S4 and S5, respectively). When we stratified the analyses by 10-year time periods, there was noticeable attenuation of mortality risk over time, mainly when using father's country of birth, but no consistent patterns were observed (Supplementary Table S6). Models which included missing categories for mother's education and father's country of birth were similar to the main analysis, but those missing father's country of birth were at higher risk compared to children with two parents born in Sweden (Supplementary Table S7).

## Discussion

This paper used total population administrative register data from Sweden to examine how all-cause mortality among children under the age of 15 varied between the children of immigrants and children with two parents born in Sweden. We identified one parental origin group with substantially and persistently higher child mortality, as compared with Swedish-born children who have two Swedish-born parents: those with parents who were born in the Middle East & Northern Africa. Similar patterns were also observed when investigating mortality for different age groups: infants, ages 1–4, and ages 5–14. Additionally, at ages 5–14, G2.0 children with mothers from Sub-Saharan Africa showed the highest mortality. In general, children with parents born in non-European or non-Western countries had higher mortality rates, but we also showed that there was considerable variation by parental country of birth, including exceptions to the general pattern of advantage for children of European-born immigrants. Children with parents born in Central & South America, Eastern Europe, and Other Western countries exhibited lower mortality, as compared with Swedish-born children of Swedish-born parents. In addition, we showed that patterns of child mortality are distinctly different for those with one Swedish-born and one foreign-born parent (G2.5). Our study therefore provides new evidence about the role of having only one foreign-born parent (i.e., G2.0 vs. G2.5), and the separate role of mothers vs. fathers across a diverse range of origins and at different ages.

As we noted in the introduction, there is a lack of prior research that has investigated under-15 mortality among the children of immigrants. Existing research has focused on infant (aged 0–1) or adult (aged 15+) mortality, largely overlooking childhood mortality.^3^ Our findings are consistent with the only prior research that appears to have studied this topic. In Denmark, higher under-5 mortality was found among children with mothers born outside Europe—particularly Turkey, Pakistan, Somalia or Iraq—and this difference persisted after controlling for disposable income.^15^ Similarly, in the Netherlands, higher under-15 mortality was found among children of immigrants with Turkish and Moroccan origins.^16^ In those studies, the most prominent causes of death were infectious diseases, congenital hereditary disorders and Sudden Infant Death Syndrome (SIDs) in infancy, hereditary disorders and external causes (e.g., accidents, drowning) at ages 1–4, and external causes at ages 5–14. Although we do not analyse causes of death, we provide descriptive information on leading causes of death by generational status and age in Supplementary Tables S8–S10, observing similar patterns across generations. Alongside SIDs, Edwards syndrome and Patau syndrome (genetic disorders caused by the presence of extra chromosomes) are leading causes of death in infancy among children born in Sweden to two parents born in Sweden, the G2, and G2.5. Furthermore, malignant neoplasms, lymphoid leukaemia, epilepsy, and exposure to forces of nature all feature prominently in these three groups at ages 1–4 and 5–14.

There are several potential explanations for our findings, which may vary for different ages and parental origin groups. First, risk factors and causes-of-death associated with infant mortality (e.g., SIDs) are less relevant (or even irrelevant) to mortality at ages 1–4 and 5–14. Second, some risks factors are more relevant for specific parental origin groups (e.g., higher rates of consanguinity, typically among couples born in the Middle East, Northern Africa, and Sub-Saharan Africa, and an increased risk of mortality from hereditary disorders). Third, age and parental origin may interact in unique ways to influence mortality risks *in addition to* separate age- and origin-specific risk factors.

A previous review identified factors most often associated with early-life mortality among the children of immigrants.^3^ These included cultural and religious practices (e.g., pre-natal screening, Caesarian sections, termination of risky pregnancies), demographic factors (e.g., maternal age), healthcare (e.g., lack of cultural sensitivity, miscommunication), maternal health (e.g., anaemia, gestational diabetes, pre-eclampsia), migration and integration (e.g., efficacy of integration policies), social determinants of health (e.g., poverty, unemployment, poor quality housing), and racism and discrimination (e.g., within the healthcare system). Many of these factors are also relevant for mortality at older childhood ages, but to varying extents and through different mechanisms.

In Sweden, a growing body of evidence supports several of these aforementioned pathways for elevated under-15 mortality among the children of immigrants. Cultural and linguistic barriers can lead to miscommunications and misunderstandings (on behalf of parents or medical practitioners, e.g., in medical procedures, risks, care plans and written health information), core information gaps, and difficulties in navigating the maternity system itself.^17^ Disparities in antenatal care use among immigrant mothers include reduced, delayed, or missed postpartum visits,^18^^,^^19^ which can increase the risk of adverse perinatal and early life mortality for their children. A greater proportion of consanguineous marriages (well documented in parts of the Middle East and North Africa and persisting to varying degrees among immigrants born in these countries) elevates the risk of autosomal recessive disorders and congenital anomalies—factors linked to elevated infant and child mortality.^20^ Female genital mutilation (for which countries in the Middle East and Northern Africa have a high prevalence^21^) is linked to higher infant mortality, through factors such as obstructed labor, neonatal asphyxia, and increased obstetric complications.^22^ Additionally, reported higher risks of fatal unintentional injuries (including transport, drowning, burns, and falls) among children of migrants in Sweden, possibly due to environmental, informational, and prevention gaps, can exacerbate mortality differentials to non-migrants during childhood.^23^ Lastly, discrimination in healthcare has been shown to undermine access, trust, and timely use of services among patients with a migrant background, including immigrant mothers.^24^^,^^25^

Infant mortality is likely driven more by ‘upstream’ factors like parental characteristics (such as education or income), cultural and religious practices, and healthcare than by individual-level mechanisms. For example, mortality at ages 1–4 may reflect pre-school conditions. Conversely, mortality at ages 5–14 may be driven more by genetic conditions, mental health, bullying, violence, or risky behaviours. Similarly, the mortality risks of immigrants' children may be impacted at any age by racism and discrimination. However, individual-level discrimination is more likely to impact older children, whereas structural racism and discriminatory policies may impact all ages, albeit (again) through different mechanisms, and to a much greater extent for some origin groups rather than others.

Prior studies have highlighted the potential role that structural racism may play in explaining health inequalities for children with parents from non-Western countries, in particular those who are members of minority groups that are frequently targets of racism and discrimination, such as visible minorities and/or refugees.^3^^,^^26^^,^^27^ In our study, this may help explain our findings for children with parents born in Asia & Oceania, Middle East & Northern Africa, or Sub-Saharan Africa. However, further research is needed, including to establish the pathways by which structural racism impacts child mortality for immigrants' children, as well as to distinguish how racism and discrimination impacts parents and children in different ways. For example, parents are more likely to face systemic barriers that make it more difficult to access the labour market or welfare system, which in turn may impact their children's living conditions and health, whereas parents and children may both be discriminated against within the healthcare system. More generally, the mechanisms through which the intergenerational transmission of inequalities occur are likely to vary for different origin groups.

The main strength of this study is its use of reliable and highly-detailed administrative data that cover the entire population of children living in Sweden, which allows us to study under-15 mortality across generations, parental countries of birth, and ages. However, this study was not without limitations. First, parental origins were grouped into broad geographic regions of the world, which masks substantial heterogeneity. They may be important because, for example, previous research found higher early-life mortality of children with mothers born in some Asian countries (specifically Sri Lanka, Bangledesh, or Pakistan), but not others (specifically Vietnam, Thailand, or India).^28^, ^29^, ^30^, ^31^, ^32^, ^33^ It may also be that certain parental origin countries (e.g., Somalia in Sub-Saharan Africa) are driving the HRs for wider geographic regions and/or that HRs do not reflect the situation for other parental origins grouped within that region. As with any analysis, our findings may also be impacted by missing data, which in our study is primarily due to missing maternal education (see Supplementary Figure S1). Despite our low level of missingness, and the results of our sensitivity analysis (which suggest a limited role of missing data), missing data may nevertheless have some impact on our findings, especially when differences between groups are relatively small. Another limitation is that there are many potential determinants of childhood mortality that are not available in our data, including those linked to aforementioned mechanisms such as discrimination. Future research could examine measures of integration and acculturation, for example by using citizenship or duration of stay. In fact, our study presents some tentative evidence that integration may explain some of the inequality we observe, at least to the extent that having one native-born parent is a measure (or proxy measure) of integration. However, further research is required to explain the extent to which parental integration is an important determinant of child mortality for immigrants’ children, and how it interacts with other social, parental, and individual-level explanations. Additional research would also be necessary to establish how generalisable our findings are beyond the Swedish context. As mentioned, some of our findings align with prior studies from Denmark and the Netherlands, but those studies do not examine many aspects that are considered here. At the same time, unlike the study in Denmark, we do not examine causes of death, which was not part of our research objectives, but is nevertheless a limitation that could be addressed by future research.

Despite these limitations, this study addresses an important gap in the literature on mortality among children of immigrants. Additional avenues for future research include the examination of further heterogeneities, which would be valuable to identify more graunlar subgroups that are at higher risk of child mortality. At the same time, future research may focus on specific groups, such as children of immigrants from specific countries, with lower socioeconomic status, or with certain health conditions. We have shown that parental origin and age (i.e. stage of childhood) are important contributors to under-15 mortality among Swedish-born children of immigrants. Our study highlights the mortality disadvantage that is experienced by some groups of second-generation children, which underscores the need for measures to help mitigate this disadvantage, in particular given the tragic consequences of any death during childhood, for individuals, families, and society.

## Contributors

All authors contributed to the conceptualisation and methodology of the study. MW curated and prepared the data, and AL conducted the formal analyses and prepared the visualisations. AL drafted the original manuscript with input from MW and BW, and all authors contributed to reviewing and editing the final manuscript, including interpreting data and results. BW and MW acquired funding for this study. All authors accessed and verified the data, approved the final version of the manuscript, and agreed to be accountable for all aspects of the work.

## Data sharing statement

The data were provided by Statistics Sweden and may not be shared with outside researchers. Similar data may be obtained after application to Statistics Sweden.

## Declaration of interests

The authors declare financial support from the organisations listed in the acknowledgements. Otherwise, AL declares no competing interests, MW declares payment for teaching and no other competing interests, and BW declares payment for reviewing funding applications and no other competing interests.
