# Supplementary material for: Mortality in children aged 0–14 years born to immigrant parents in Sweden: a total-population cohort study
Source: Lancet Reg Health Eur. 2026 Apr 2;65:101666. doi: 10.1016/j.lanepe.2026.101666 (PMC13084368; doi:10.1016/j.lanepe.2026.101666)
Supplement: Supplementary Material [file mmc1.docx]

Supplementary Material

Mortality in children aged 0–15 years born to immigrant parents in Sweden: a total-population cohort study

Table of Contents

[Study population inclusion and exclusion flow diagram 2](#_Toc219996324)

[Minimally adjusted models 3](#_Toc219996325)

[Estimates by age group and parental origin, separately for mothers and fathers 4](#_Toc219996326)

[Sensitivity analysis: Stratified by generation 5](#_Toc219996327)

[Sensitivity analysis: Parental refugee status 6](#_Toc219996328)

[Sensitivity analysis: Father’s education 7](#_Toc219996329)

[Sensitivity analysis: Both parents’ education 8](#_Toc219996330)

[Sensitivity analysis: 10-year period trends 9](#_Toc219996331)

[Sensitivity analysis: Include missing category for mother’s education and father’s country of birth 10](#_Toc219996332)

[Cause of Death Descriptive Statistics 11](#_Toc219996333)

# **Study population inclusion and exclusion flow diagram**

**Figure S1.** Study population inclusion and exclusion flow diagram

# **Minimally adjusted models**

******

**Figure S2.** Minimally adjusted hazard ratios of under-15 mortality among the children of immigrants according to generational status and the migration background of the parents, 1990-2019. Note. Models are adjusted for sex, birth year, birth order, and maternal age. The reference group are children born in Sweden to two parents born in Sweden.

# **Estimates by age group and parental origin, separately for mothers and fathers for the G2.0**

**Table S1.** Hazard ratios and 95% confidence intervals of under-15 mortality among the children of immigrants, by generation, age group, and parental country of birth, 1990-2019.

|  |  | **Mother’s country of birth** | | | **Father’s country of birth** | | |
| --- | --- | --- | --- | --- | --- | --- | --- |
|  |  | Infant | Ages 1-4 | Ages 5-14 | Infant | Ages 1-4 | Ages 5-14 |
| **G2.0**  **(both parents born abroad)** | Two parents born in Sweden (Reference) | 1·00 | 1·00 | 1·00 | 1·00 | 1·00 | 1·00 |
|  | Central & South America | 0·59 (0·44–0·81) | 0·56 (0·25–1·26) | 1·05 (0·58–1·90) | 0·62 (0·45–0·84) | 0·58 (0·26–1·30) | 0·88 (0·46–1·71) |
|  | Eastern Europe | 0·72 (0·57–0·91) | 0·35 (0·15–0·85) | 1·04 (0·60–1·80) | 0·76 (0·59–0·99) | 0·37 (0·14–0·99) | 1·08 (0·58–2·02) |
|  | Other Western | 0·82 (0·71–0·95) | 1·02 (0·73–1·44) | 1·04 (0·74–1·45) | 0·79 (0·69–0·92) | 1·05 (0·75–1·47) | 1·06 (0·76–1·47) |
|  | Other Nordic | 0·90 (0·60–1·36) | 1·50 (0·62–3·63) | 0·70 (0·17–2·80) | 1·09 (0·75–1·58) | 0·60 (0·15–2·39) | 0·00 (0·00–Inf) |
|  | Asia and Oceania | 0·90 (0·78–1·04) | 1·27 (0·91–1·78) | 1·17 (0·82–1·66) | 0·89 (0·77–1·04) | 1·15 (0·81–1·64) | 1·14 (0·80–1·63) |
|  | Sub-Saharan Africa | 0·83 (0·72–0·96) | 0·96 (0·67–1·38) | 1·80 (1·28–2·52) | 0·82 (0·71–0·94) | 0·99 (0·69–1·40) | 1·76 (1·26–2·45) |
|  | Finland | 1·06 (0·81–1·37) | 1·15 (0·59–2·22) | 0·52 (0·21–1·24) | 1·11 (0·83–1·47) | 1·30 (0·65–2·61) | 0·64 (0·27–1·55) |
|  | Middle East & Northern Africa | 1·10 (1·00–1·22) | 1·67 (1·34–2·07) | 1·35 (1·07–1·69) | 1·08 (0·99–1·19) | 1·65 (1·33–2·04) | 1·35 (1·08–1·68) |
| **Sex** | Male (Reference) | 1·00 | 1·00 | 1·00 | 1·00 | 1·00 | 1·00 |
|  | Female | 0·82 (0·78–0·85) | 0·88 (0·79–0·99) | 0·85 (0·77–0·94) | 0·82 (0·78–0·85) | 0·88 (0·79–0·99) | 0·85 (0·77–0·94) |
| **Birth order** |  | 1·00 (0·98–1·03) | 1·01 (0·95–1·07) | 0·97 (0·92–1·03) | 1·00 (0·98–1·03) | 1·01 (0·95–1·07) | 0·97 (0·92–1·03) |
| **Birth year** |  | 0·97 (0·97–0·97) | 0·98 (0·97–0·99) | 0·97 (0·96–0·98) | 0·97 (0·97–0·97) | 0·98 (0·97–0·99) | 0·97 (0·96–0·98) |
| **Maternal age** |  | 1·01 (1·01–1·02) | 1·00 (0·99–1·01) | 1·00 (0·99–1·02) | 1·01 (1·01–1·02) | 1·00 (0·99–1·01) | 1·00 (0·99–1·02) |
| **Residence** | Urban (Reference) | 1·00 | 1·00 | 1·00 | 1·00 | 1·00 | 1·00 |
|  | Rural | 1·29 (1·23–1·35) | 1·11 (0·99–1·24) | 1·21 (1·09–1·34) | 1·29 (1·23–1·35) | 1·11 (0·99–1·24) | 1·21 (1·09–1·34) |
| **Marital status** | Married or partnered (Reference) | 1·00 | 1·00 | 1·00 | 1·00 | 1·00 | 1·00 |
|  | Cohabiting | 0·42 (0·40–0·45) | 0·69 (0·60–0·80) | 0·79 (0·69–0·90) | 0·42 (0·40–0·45) | 0·69 (0·60–0·80) | 0·79 (0·69–0·90) |
|  | Single | 2·27 (2·11–2·44) | 2·92 (2·44–3·48) | 1·26 (1·08–1·47) | 2·26 (2·10–2·43) | 2·89 (2·42–3·46) | 1·25 (1·07–1·46) |
| **Mother’s education** | Low (Reference) | 1·00 | 1·00 | 1·00 | 1·00 | 1·00 | 1·00 |
|  | Medium | 0·91 (0·85–0·97) | 0·94 (0·81–1·10) | 0·82 (0·71–0·95) | 0·91 (0·85–0·97) | 0·94 (0·80–1·10) | 0·82 (0·71–0·95) |
|  | High | 0·78 (0·73–0·85) | 0·80 (0·67–0·97) | 0·76 (0·64–0·90) | 0·78 (0·72–0·84) | 0·80 (0·67–0·96) | 0·76 (0·64–0·90) |
| **Household disposable income quintile** | Lowest (Reference) | 1·00 | 1·00 | 1·00 | 1·00 | 1·00 | 1·00 |
|  | Lower | 0·81 (0·76–0·87) | 1·14 (0·95–1·37) | 1·15 (0·98–1·35) | 0·81 (0·76–0·87) | 1·14 (0·95–1·36) | 1·15 (0·98–1·35) |
|  | Middle | 0·80 (0·74–0·87) | 0·92 (0·74–1·14) | 0·76 (0·62–0·92) | 0·80 (0·74–0·87) | 0·91 (0·74–1·13) | 0·76 (0·62–0·91) |
|  | Higher | 0·82 (0·75–0·89) | 1·07 (0·86–1·34) | 0·82 (0·68–0·99) | 0·82 (0·75–0·89) | 1·07 (0·85–1·33) | 0·82 (0·67–0·99) |
|  | Highest | 0·85 (0·78–0·94) | 1·23 (0·98–1·55) | 1·03 (0·84–1·25) | 0·85 (0·77–0·93) | 1·23 (0·98–1·54) | 1·03 (0·84–1·25) |

# **Sensitivity analysis: Stratified by generation**

**Table S2.** Hazard ratios and 95% confidence intervals of under-15 mortality among the children of immigrants stratified by generation, by parental country of birth, 1990-2019.

|  |  | **Mother’s country of birth** | | **Father’s country of birth** | |
| --- | --- | --- | --- | --- | --- |
|  |  | G2.0 | G2.5 | G2.0 | G2.5 |
| **Country of birth** | Two parents born in Sweden (Reference) | 1·00 | 1·00 | 1·00 | 1·00 |
|  | Sweden^a^ | ·· | 0·80 (0·74–0·86) | ·· | 1·02 (0·95–1·11) |
|  | Central & South America | 0·66 (0·51–0·86) | 0·99 (0·77–1·26) | 0·66 (0·51–0·86) | 0·59 (0·45–0·77) |
|  | Eastern Europe | 0·72 (0·59–0·89) | 1·07 (0·86–1·34) | 0·76 (0·60–0·97) | 0·73 (0·51–1·04) |
|  | Other Western | 0·88 (0·78–1·00) | 0·99 (0·83–1·18) | 0·87 (0·77–0·99) | 0·84 (0·74–0·96) |
|  | Other Nordic | 0·96 (0·67–1·37) | 1·14 (0·92–1·41) | 0·95 (0·66–1·35) | 1·06 (0·87–1·30) |
|  | Asia and Oceania | 0·99 (0·87–1·12) | 1·01 (0·87–1·18) | 0·97 (0·85–1·10) | 0·78 (0·62–0·98) |
|  | Sub-Saharan Africa | 0·96 (0·85–1·09) | 0·63 (0·38–1·02) | 0·95 (0·84–1·07) | 0·47 (0·33–0·68) |
|  | Finland | 1·01 (0·80–1·27) | 1·16 (0·99–1·37) | 1·08 (0·84–1·39) | 0·84 (0·69–1·02) |
|  | Middle East & Northern Africa | 1·22 (1·12–1·32) | 0·65 (0·42–1·00) | 1·20 (1·11–1·30) | 0·81 (0·67–0·97) |
| **Sex** | Male (Reference) | 1·00 | 1·00 | 1·00 | 1·00 |
|  | Female | 0·83 (0·80–0·86) | 0·82 (0·79–0·85) | 0·83 (0·80–0·86) | 0·82 (0·79–0·85) |
| **Birth order** |  | 0·99 (0·97–1·02) | 0·98 (0·96–1·00) | 1·00 (0·97–1·02) | 0·98 (0·96–1·01) |
| **Birth year** |  | 0·97 (0·97–0·97) | 0·97 (0·97–0·97) | 0·97 (0·97–0·97) | 0·97 (0·97–0·97) |
| **Maternal age** |  | 1·01 (1·01–1·02) | 1·01 (1·01–1·02) | 1·01 (1·01–1·02) | 1·01 (1·01–1·02) |
| **Residence** | Urban (Reference) | 1·00 | 1·00 | 1·00 | 1·00 |
|  | Rural | 1·25 (1·20–1·30) | 1·29 (1·24–1·34) | 1·25 (1·20–1·30) | 1·29 (1·24–1·34) |
| **Marital status** | Married or partnered (Reference) | 1·00 | 1·00 | 1·00 | 1·00 |
|  | Cohabiting | 0·48 (0·45–0·50) | 0·48 (0·46–0·51) | 0·48 (0·45–0·50) | 0·48 (0·46–0·51) |
|  | Single | 2·09 (1·97–2·22) | 2·36 (2·21–2·51) | 2·09 (1·96–2·22) | 2·37 (2·22–2·53) |
| **Mother’s education** | Low (Reference) | 1·00 | 1·00 | 1·00 | 1·00 |
|  | Medium | 0·89 (0·84–0·94) | 0·92 (0·87–0·98) | 0·89 (0·84–0·94) | 0·92 (0·87–0·98) |
|  | High | 0·77 (0·72–0·82) | 0·80 (0·74–0·86) | 0·76 (0·72–0·82) | 0·80 (0·75–0·86) |
| **Household disposable income quintile** | Lowest (Reference) | 1·00 | 1·00 | 1·00 | 1·00 |
|  | Lower | 0·88 (0·83–0·94) | 0·89 (0·84–0·96) | 0·88 (0·83–0·94) | 0·89 (0·84–0·95) |
|  | Middle | 0·83 (0·77–0·89) | 0·86 (0·80–0·93) | 0·82 (0·77–0·89) | 0·86 (0·80–0·93) |
|  | Higher | 0·87 (0·81–0·94) | 0·91 (0·84–0·98) | 0·87 (0·80–0·94) | 0·91 (0·84–0·98) |
|  | Highest | 0·95 (0·88–1·03) | 1·00 (0·93–1·09) | 0·95 (0·88–1·03) | 1·00 (0·93–1·09) |

*^a^* If one parent was born in Sweden, the other parent was born abroad

# **Sensitivity analysis: Parental refugee status**

**Table S3.** Hazard ratios and 95% confidence intervals of under-15 mortality among the children of immigrants, by generation and parental refugee status, 1990-2019.

|  |  | **Model 0** | **Model 1a** | **Model 1b** | **Model 1c** |
| --- | --- | --- | --- | --- | --- |
| **Generation and parental refugee status** | Two parents born in Sweden (Reference) | 1·00 | 1·00 | 1·00 | 1·00 |
|  | G2.0, no refugee parent | 1·33 (1·24–1·43) | 1·11 (1·03–1·20) | 1·07 (0·99–1·15) | 1·03 (0·96–1·12) |
|  | G2.5, no refugee parent | 1·06 (1·00–1·13) | 0·94 (0·88–0·99) | 0·93 (0·87–0·98) | 0·91 (0·86–0·97) |
|  | Swedish-born to two Swedish born parents, refugee parent | 0·87 (0·22–3·49) | 0·65 (0·16–2·60) | 0·63 (0·16–2·53) | 0·63 (0·16–2·51) |
|  | G2.0, refugee parent | 1·41 (1·32–1·51) | 1·10 (1·03–1·18) | 1·04 (0·97–1·12) | 1·00 (0·93–1·07) |
|  | G2.5, refugee parent | 1·01 (0·84–1·22) | 0·78 (0·65–0·93) | 0·76 (0·64–0·92) | 0·75 (0·63–0·90) |
| **Sex** | Male (reference) | 1·00 | 1·00 | 1·00 | 1·00 |
|  | Female | 0·82 (0·79–0·85) | 0·82 (0·79–0·85) | 0·82 (0·79–0·85) | 0·82 (0·79–0·85) |
| **Birth order** |  | 1·07 (1·05–1·09) | 1·02 (1·00–1·04) | 0·99 (0·97–1·01) | 1·00 (0·98–1·02) |
| **Birth year** |  | 0·97 (0·96–0·97) | 0·97 (0·97–0·97) | 0·97 (0·97–0·97) | 0·97 (0·97–0·97) |
| **Maternal age** |  | 1·00 (1·00–1·01) | 1·00 (1·00–1·01) | 1·01 (1·01–1·02) | 1·01 (1·01–1·02) |
| **Residence** | Urban (Reference) |  | 1·00 | 1·00 | 1·00 |
|  | Rural |  | 1·26 (1·22–1·31) | 1·26 (1·21–1·30) | 1·26 (1·21–1·31) |
| **Marital status** | Married or partnered (Reference) |  | 1·00 | 1·00 | 1·00 |
|  | Cohabiting |  | 0·47 (0·45–0·50) | 0·46 (0·44–0·49) | 0·47 (0·44–0·49) |
|  | Single |  | 2·23 (2·13–2·33) | 2·12 (2·02–2·22) | 1·96 (1·85–2·07) |
| **Mother’s education** | Low (Reference) |  | ·· | 1·00 | 1·00 |
|  | Medium |  | ·· | 0·87 (0·83–0·92) | 0·88 (0·83–0·93) |
|  | High |  | ·· | 0·75 (0·71–0·80) | 0·76 (0·71–0·81) |
| **Household disposable income quintile** | Lowest (Reference) |  | ·· | ·· | 1·00 |
|  | Lower |  | ·· | ·· | 0·89 (0·84–0·94) |
|  | Middle |  | ·· | ·· | 0·83 (0·77–0·88) |
|  | Higher |  | ·· | ·· | 0·87 (0·81–0·93) |
|  | Highest |  | ·· | ·· | 0·95 (0·88–1·02) |

# **Sensitivity analysis: Father’s education**

**Table S4.** Hazard ratios and 95% confidence intervals of under-15 mortality among the children of immigrants using father’s education, by generation and parental country of birth, 1990-2019.

|  |  | **Mother’s country of birth** | | **Father’s country of birth** | |
| --- | --- | --- | --- | --- | --- |
|  |  | Model 1a | Model 1b | Model 2a | Model 2b |
| **G2.0**  **(Both parents born abroad)** | Two parents born in Sweden (Reference) | 1·00 | 1·00 | 1·00 | 1·00 |
|  | Central & South America | 0·67 (0·50–0·88) | 0·65 (0·49–0·86) | 0·65 (0·49–0·87) | 0·64 (0·48–0·85) |
|  | Eastern Europe | 0·74 (0·59–0·92) | 0·72 (0·57–0·90) | 0·80 (0·62–1·02) | 0·78 (0·61–1·00) |
|  | Other Western | 0·94 (0·83–1·07) | 0·92 (0·81–1·04) | 0·94 (0·83–1·07) | 0·92 (0·81–1·04) |
|  | Other Nordic | 1·14 (0·79–1·63) | 1·10 (0·77–1·58) | 0·98 (0·66–1·45) | 0·95 (0·64–1·40) |
|  | Asia and Oceania | 1·09 (0·96–1·24) | 1·05 (0·92–1·19) | 1·06 (0·93–1·21) | 1·02 (0·90–1·16) |
|  | Sub-Saharan Africa | 1·13 (1·00–1·29) | 1·10 (0·97–1·25) | 1·11 (0·98–1·26) | 1·08 (0·95–1·22) |
|  | Finland | 1·08 (0·85–1·38) | 1·07 (0·84–1·36) | 1·14 (0·88–1·48) | 1·13 (0·87–1·47) |
|  | Middle East & Northern Africa | 1·32 (1·22–1·43) | 1·26 (1·16–1·37) | 1·31 (1·21–1·42) | 1·25 (1·15–1·35) |
| **G2.5**  **(One parent born abroad)** | Two parents born in Sweden (Reference) | 1·00 | 1·00 | 1·00 | 1·00 |
|  | Sweden^a^ | 0·86 (0·80–0·93) | 0·85 (0·79–0·92) | 1·04 (0·96–1·12) | 1·03 (0·95–1·11) |
|  | Central & South America | 1·01 (0·79–1·29) | 1·00 (0·78–1·28) | 0·64 (0·49–0·84) | 0·63 (0·48–0·83) |
|  | Eastern Europe | 1·07 (0·85–1·34) | 1·06 (0·85–1·33) | 0·75 (0·52–1·08) | 0·74 (0·51–1·07) |
|  | Other Western | 0·99 (0·83–1·19) | 0·98 (0·82–1·17) | 0·95 (0·83–1·10) | 0·94 (0·82–1·08) |
|  | Other Nordic | 1·16 (0·94–1·44) | 1·16 (0·93–1·43) | 1·11 (0·90–1·38) | 1·10 (0·89–1·37) |
|  | Asia and Oceania | 1·03 (0·89–1·21) | 1·02 (0·88–1·19) | 0·87 (0·69–1·10) | 0·86 (0·68–1·08) |
|  | Sub-Saharan Africa | 0·66 (0·40–1·07) | 0·65 (0·40–1·06) | 0·53 (0·36–0·78) | 0·52 (0·35–0·76) |
|  | Finland | 1·17 (1·00–1·38) | 1·17 (0·99–1·38) | 0·85 (0·70–1·04) | 0·85 (0·69–1·04) |
|  | Middle East & Northern Africa | 0·66 (0·42–1·02) | 0·65 (0·42–1·00) | 0·88 (0·73–1·06) | 0·86 (0·71–1·03) |
| **Sex** | Male (Reference) | 1·00 | 1·00 | 1·00 | 1·00 |
|  | Female | 0·82 (0·79–0·85) | 0·82 (0·79–0·85) | 0·82 (0·79–0·85) | 0·82 (0·79–0·85) |
| **Birth order** |  | 1·00 (0·98–1·02) | 1·00 (0·98–1·02) | 1·00 (0·98–1·02) | 1·00 (0·98–1·02) |
| **Birth year** |  | 0·97 (0·97–0·97) | 0·97 (0·97–0·97) | 0·97 (0·97–0·97) | 0·97 (0·97–0·97) |
| **Maternal age** |  | 1·01 (1·01–1·01) | 1·01 (1·01–1·01) | 1·01 (1·01–1·01) | 1·01 (1·01–1·01) |
| **Residence** | Urban (Reference) | 1·00 | 1·00 | 1·00 | 1·00 |
|  | Rural | 1·24 (1·20–1·29) | 1·25 (1·20–1·30) | 1·24 (1·20–1·29) | 1·25 (1·20–1·30) |
| **Marital status** | Married or partnered (Reference) | 1·00 | 1·00 | 1·00 | 1·00 |
|  | Cohabiting | 0·47 (0·45–0·49) | 0·47 (0·45–0·50) | 0·47 (0·45–0·49) | 0·47 (0·45–0·50) |
|  | Single | 2·27 (2·17–2·38) | 2·10 (1·98–2·22) | 2·28 (2·18–2·39) | 2·11 (1·99–2·23) |
| **Father’s education** | Low (Reference) | 1·00 | 1·00 | 1·00 | 1·00 |
|  | Medium | 0·93 (0·89–0·98) | 0·94 (0·90–0·99) | 0·93 (0·89–0·98) | 0·94 (0·90–0·99) |
|  | High | 0·81 (0·77–0·86) | 0·81 (0·77–0·86) | 0·81 (0·77–0·86) | 0·81 (0·77–0·86) |
| **Household disposable income quintile** | Lowest (Reference) | ·· | 1·00 | ·· | 1·00 |
|  | Lower | ·· | 0·89 (0·84–0·94) | ·· | 0·89 (0·84–0·94) |
|  | Middle | ·· | 0·83 (0·78–0·89) | ·· | 0·83 (0·78–0·89) |
|  | Higher | ·· | 0·87 (0·81–0·94) | ·· | 0·87 (0·81–0·94) |
|  | Highest | ·· | 0·96 (0·89–1·04) | ·· | 0·96 (0·89–1·04) |

*^a^* If one parent was born in Sweden, the other parent was born abroad

# **Sensitivity analysis: Both parents’ education**

**Table S5.** Hazard ratios and 95% confidence intervals of under-15 mortality among the children of immigrants using both parents’ education, by generation and parental country of birth, 1990-2019.

|  |  | **Mother’s country of birth** | | **Father’s country of birth** | |
| --- | --- | --- | --- | --- | --- |
|  |  | Model 1a | Model 1b | Model 2a | Model 2b |
| **G2.0**  **(Both parents born abroad)** | Two parents born in Sweden (Reference) | 1·00 | 1·00 | 1·00 | 1·00 |
|  | Central & South America | 0·65 (0·49–0·86) | 0·64 (0·49–0·85) | 0·64 (0·48–0·85) | 0·63 (0·48–0·84) |
|  | Eastern Europe | 0·74 (0·59–0·92) | 0·72 (0·58–0·90) | 0·80 (0·62–1·02) | 0·78 (0·61–1·00) |
|  | Other Western | 0·92 (0·81–1·05) | 0·90 (0·79–1·03) | 0·92 (0·81–1·05) | 0·90 (0·79–1·02) |
|  | Other Nordic | 1·13 (0·79–1·61) | 1·10 (0·77–1·57) | 0·97 (0·66–1·44) | 0·95 (0·64–1·40) |
|  | Asia and Oceania | 1·06 (0·93–1·20) | 1·03 (0·90–1·17) | 1·03 (0·91–1·18) | 1·00 (0·88–1·14) |
|  | Sub-Saharan Africa | 1·08 (0·95–1·23) | 1·05 (0·93–1·20) | 1·06 (0·94–1·21) | 1·04 (0·91–1·18) |
|  | Finland | 1·07 (0·84–1·36) | 1·06 (0·83–1·35) | 1·12 (0·86–1·45) | 1·11 (0·86–1·44) |
|  | Middle East & Northern Africa | 1·28 (1·18–1·38) | 1·23 (1·13–1·33) | 1·27 (1·17–1·37) | 1·22 (1·12–1·32) |
| **G2.5**  **(One parent born abroad)** | Two parents born in Sweden (Reference) | 1·00 | 1·00 | 1·00 | 1·00 |
|  | Sweden | 0·86 (0·79–0·93) | 0·85 (0·78–0·92) | 1·03 (0·95–1·11) | 1·02 (0·95–1·11) |
|  | Central & South America | 1·00 (0·78–1·28) | 0·99 (0·78–1·27) | 0·64 (0·49–0·84) | 0·63 (0·48–0·83) |
|  | Eastern Europe | 1·08 (0·86–1·35) | 1·07 (0·85–1·34) | 0·74 (0·51–1·07) | 0·73 (0·51–1·06) |
|  | Other Western | 1·00 (0·83–1·19) | 0·99 (0·83–1·18) | 0·95 (0·83–1·09) | 0·94 (0·82–1·08) |
|  | Other Nordic | 1·15 (0·93–1·43) | 1·15 (0·93–1·42) | 1·11 (0·89–1·37) | 1·10 (0·88–1·36) |
|  | Asia and Oceania | 1·01 (0·87–1·18) | 1·01 (0·86–1·18) | 0·86 (0·68–1·09) | 0·85 (0·67–1·08) |
|  | Sub-Saharan Africa | 0·65 (0·40–1·05) | 0·64 (0·39–1·05) | 0·52 (0·35–0·78) | 0·51 (0·35–0·76) |
|  | Finland | 1·17 (0·99–1·38) | 1·16 (0·99–1·37) | 0·84 (0·69–1·03) | 0·84 (0·69–1·03) |
|  | Middle East & Northern Africa | 0·64 (0·41–1·00) | 0·64 (0·41–0·99) | 0·86 (0·71–1·04) | 0·84 (0·70–1·02) |
| **Sex** | Male (Reference) | 1·00 | 1·00 | 1·00 | 1·00 |
|  | Female | 0·82 (0·79–0·85) | 0·82 (0·79–0·85) | 0·82 (0·79–0·85) | 0·82 (0·79–0·85) |
| **Birth order** |  | 0·98 (0·97–1·00) | 0·99 (0·97–1·01) | 0·99 (0·97–1·00) | 0·99 (0·97–1·01) |
| **Birth year** |  | 0·97 (0·97–0·97) | 0·97 (0·97–0·97) | 0·97 (0·97–0·97) | 0·97 (0·97–0·97) |
| **Maternal age** |  | 1·01 (1·01–1·02) | 1·01 (1·01–1·02) | 1·01 (1·01–1·02) | 1·01 (1·01–1·02) |
| **Residence** | Urban (Reference) | 1·00 | 1·00 | 1·00 | 1·00 |
|  | Rural | 1·24 (1·20–1·29) | 1·25 (1·20–1·30) | 1·24 (1·20–1·29) | 1·25 (1·20–1·30) |
| **Marital status** | Married or partnered (Reference) | 1·00 | 1·00 | 1·00 | 1·00 |
|  | Cohabiting | 0·46 (0·44–0·49) | 0·47 (0·45–0·49) | 0·47 (0·44–0·49) | 0·47 (0·45–0·49) |
|  | Single | 2·21 (2·11–2·32) | 2·06 (1·95–2·19) | 2·22 (2·12–2·33) | 2·07 (1·95–2·19) |
| **Mother’s education** | Low (Reference) | 1·00 | 1·00 | 1·00 | 1·00 |
|  | Medium | 0·90 (0·85–0·95) | 0·91 (0·86–0·96) | 0·90 (0·85–0·95) | 0·91 (0·86–0·96) |
|  | High | 0·81 (0·76–0·86) | 0·82 (0·77–0·87) | 0·81 (0·76–0·86) | 0·82 (0·76–0·87) |
| **Father’s education** | Low (Reference) | 1·00 | 1·00 | 1·00 | 1·00 |
|  | Medium | 0·95 (0·91–1·00) | 0·96 (0·91–1·01) | 0·95 (0·91–1·00) | 0·96 (0·91–1·01) |
|  | High | 0·86 (0·81–0·92) | 0·86 (0·81–0·92) | 0·86 (0·81–0·92) | 0·86 (0·81–0·92) |
| **Household disposable income quintile** | Lowest (Reference) | ·· | 1·00 | ·· | 1·00 |
|  | Lower | ·· | 0·89 (0·84–0·95) | ·· | 0·89 (0·84–0·95) |
|  | Middle | ·· | 0·84 (0·79–0·90) | ·· | 0·84 (0·79–0·90) |
|  | Higher | ·· | 0·89 (0·83–0·95) | ·· | 0·89 (0·83–0·95) |
|  | Highest | ·· | 0·98 (0·91–1·06) | ·· | 0·98 (0·91–1·06) |

*^a^* If one parent was born in Sweden, the other parent was born abroad

# **Sensitivity analysis: 10-year period trends**

**Table S6.** Hazard ratios and 95% confidence intervals of under-15 mortality among the children of immigrants, by generation, parental country of birth, and 10-year periods, 1990-2019.

|  |  | **Mother’s country of birth** | | | **Father’s country of birth** | | |
| --- | --- | --- | --- | --- | --- | --- | --- |
|  |  | 1990-1999 | 2000-2009 | 2010-2019 | 1990-1999 | 2000-2009 | 2010-2019 |
| **G2.0**  **(Both parents born abroad)** | Two parents born in Sweden (Reference) | 1·00 | 1·00 | 1·00 | 1·00 | 1·00 | 1·00 |
|  | Central & South America | 0·71 (0·48–1·03) | 0·65 (0·41–1·02) | 0·61 (0·35–1·08) | 0·71 (0·49–1·04) | 0·66 (0·41–1·05) | 0·60 (0·33–1·08) |
|  | Eastern Europe | 0·70 (0·47–1·05) | 0·57 (0·37–0·88) | 0·74 (0·55–1·00) | 0·86 (0·57–1·31) | 0·58 (0·35–0·97) | 0·72 (0·51–1·01) |
|  | Other Western | 1·02 (0·82–1·27) | 0·86 (0·70–1·06) | 0·74 (0·59–0·93) | 0·97 (0·79–1·21) | 0·85 (0·69–1·04) | 0·75 (0·60–0·94) |
|  | Other Nordic | 0·83 (0·48–1·44) | 1·04 (0·54–2·00) | 0·98 (0·49–1·96) | 0·96 (0·57–1·62) | 0·53 (0·22–1·28) | 1·21 (0·67–2·19) |
|  | Asia and Oceania | 0·96 (0·76–1·20) | 0·92 (0·73–1·16) | 0·94 (0·76–1·15) | 0·91 (0·72–1·14) | 0·94 (0·75–1·18) | 0·92 (0·75–1·14) |
|  | Sub-Saharan Africa | 1·19 (0·92–1·54) | 0·95 (0·76–1·20) | 0·73 (0·60–0·88) | 1·16 (0·90–1·50) | 0·97 (0·77–1·21) | 0·71 (0·59–0·85) |
|  | Finland | 1·21 (0·95–1·56) | 0·64 (0·32–1·29) | 0·26 (0·04–1·84) | 1·30 (0·99–1·71) | 0·47 (0·19–1·12) | 0·82 (0·26–2·54) |
|  | Middle East & Northern Africa | 1·26 (1·09–1·46) | 1·26 (1·09–1·44) | 0·97 (0·85–1·12) | 1·22 (1·06–1·41) | 1·24 (1·08–1·42) | 0·98 (0·85–1·12) |
| **G2.5**  **(One parent born abroad)** | Two parents born in Sweden (Reference) | 1·00 | 1·00 | 1·00 | 1·00 | 1·00 | 1·00 |
|  | Sweden | 0·93 (0·83–1·04) | 0·79 (0·69–0·90) | 0·61 (0·52–0·72) | 1·14 (1·02–1·28) | 0·89 (0·77–1·02) | 0·95 (0·81–1·11) |
|  | Central & South America | 0·95 (0·60–1·51) | 1·05 (0·72–1·52) | 0·88 (0·56–1·38) | 0·42 (0·24–0·74) | 0·63 (0·42–0·96) | 0·70 (0·45–1·07) |
|  | Eastern Europe | 1·27 (0·89–1·80) | 0·77 (0·49–1·19) | 1·15 (0·77–1·71) | 1·16 (0·72–1·86) | 0·70 (0·38–1·31) | 0·29 (0·11–0·78) |
|  | Other Western | 1·25 (0·97–1·61) | 0·77 (0·54–1·09) | 0·83 (0·58–1·18) | 0·91 (0·74–1·11) | 0·90 (0·72–1·12) | 0·67 (0·50–0·90) |
|  | Other Nordic | 1·26 (0·96–1·65) | 0·98 (0·64–1·50) | 1·02 (0·55–1·89) | 1·34 (1·04–1·73) | 0·90 (0·60–1·35) | 0·58 (0·30–1·12) |
|  | Asia and Oceania | 1·00 (0·74–1·36) | 0·94 (0·74–1·21) | 1·01 (0·78–1·30) | 0·87 (0·59–1·28) | 0·83 (0·56–1·22) | 0·67 (0·44–1·02) |
|  | Sub-Saharan Africa | 0·77 (0·32–1·84) | 0·81 (0·40–1·61) | 0·30 (0·10–0·92) | 0·72 (0·41–1·27) | 0·55 (0·31–0·98) | 0·21 (0·09–0·50) |
|  | Finland | 1·18 (0·97–1·44) | 0·98 (0·67–1·44) | 1·64 (1·00–2·69) | 1·04 (0·83–1·29) | 0·59 (0·37–0·95) | 0·11 (0·02–0·81) |
|  | Middle East & Northern Africa | 0·35 (0·09–1·39) | 0·51 (0·23–1·15) | 0·76 (0·43–1·34) | 0·69 (0·48–0·98) | 0·86 (0·64–1·15) | 0·78 (0·58–1·05) |
| **Sex** | Male (Reference) | 1·00 | 1·00 | 1·00 | 1·00 | 1·00 | 1·00 |
|  | Female | 0·78 (0·74–0·82) | 0·88 (0·82–0·94) | 0·83 (0·77–0·90) | 0·78 (0·74–0·82) | 0·88 (0·82–0·94) | 0·83 (0·77–0·90) |
| **Birth order** |  | 1·00 (0·97–1·03) | 0·96 (0·93–1·00) | 1·02 (0·98–1·06) | 1·00 (0·98–1·03) | 0·97 (0·93–1·00) | 1·02 (0·98–1·06) |
| **Birth year** |  | 0·95 (0·94–0·96) | 0·96 (0·95–0·97) | 0·98 (0·96–0·99) | 0·95 (0·94–0·96) | 0·96 (0·95–0·97) | 0·98 (0·96–0·99) |
| **Maternal age** |  | 1·01 (1·01–1·02) | 1·01 (1·01–1·02) | 1·02 (1·01–1·02) | 1·01 (1·01–1·02) | 1·01 (1·01–1·02) | 1·02 (1·01–1·02) |
| **Residence** | Urban (Reference) | 1·00 | 1·00 | 1·00 | 1·00 | 1·00 | 1·00 |
|  | Rural | 1·18 (1·12–1·25) | 1·33 (1·24–1·42) | 1·24 (1·15–1·34) | 1·18 (1·12–1·25) | 1·33 (1·24–1·42) | 1·25 (1·15–1·35) |
| **Marital status** | Married or partnered (Reference) | 1·00 | 1·00 | 1·00 | 1·00 | 1·00 | 1·00 |
|  | Cohabiting | 0·60 (0·56–0·65) | 0·38 (0·34–0·41) | 0·35 (0·32–0·39) | 0·60 (0·56–0·64) | 0·38 (0·35–0·42) | 0·35 (0·32–0·40) |
|  | Single | 1·52 (1·39–1·67) | 2·43 (2·20–2·69) | 2·33 (2·09–2·60) | 1·52 (1·39–1·67) | 2·44 (2·21–2·69) | 2·34 (2·10–2·61) |
| **Mother’s education** | Low (Reference) | 1·00 | 1·00 | 1·00 | 1·00 | 1·00 | 1·00 |
|  | Medium | 0·83 (0·77–0·89) | 1·00 (0·90–1·11) | 0·93 (0·82–1·04) | 0·83 (0·77–0·89) | 1·00 (0·90–1·10) | 0·92 (0·82–1·04) |
|  | High | 0·72 (0·66–0·79) | 0·86 (0·77–0·96) | 0·84 (0·74–0·95) | 0·72 (0·66–0·79) | 0·86 (0·76–0·96) | 0·83 (0·73–0·95) |
| **Household disposable income quintile** | Lowest (Reference) | 1·00 | 1·00 | 1·00 | 1·00 | 1·00 | 1·00 |
|  | Lower | 0·99 (0·91–1·09) | 0·87 (0·79–0·96) | 0·76 (0·68–0·85) | 0·99 (0·90–1·08) | 0·87 (0·79–0·96) | 0·76 (0·68–0·85) |
|  | Middle | 0·91 (0·83–1·01) | 0·82 (0·72–0·92) | 0·65 (0·57–0·75) | 0·91 (0·82–1·00) | 0·82 (0·72–0·92) | 0·65 (0·57–0·75) |
|  | Higher | 0·97 (0·88–1·07) | 0·82 (0·72–0·94) | 0·61 (0·52–0·73) | 0·96 (0·87–1·06) | 0·83 (0·72–0·94) | 0·62 (0·52–0·73) |
|  | Highest | 1·09 (0·98–1·20) | 0·84 (0·73–0·96) | 0·63 (0·52–0·76) | 1·08 (0·97–1·19) | 0·84 (0·73–0·97) | 0·63 (0·53–0·76) |

*^a^* If one parent was born in Sweden, the other parent was born abroad

# **Sensitivity analysis: Include missing category for mother’s education and father’s country of birth**

**Table S7.** Hazard ratios and 95% confidence intervals of under-15 mortality among the children of immigrants using missing categories in mother’s education and father’s country of birth, by generation and parental country of birth, 1990-2019.

|  |  | **Mother’s country of birth** | **Father’s country of birth** |
| --- | --- | --- | --- |
| **G2.0**  **(Both parents born abroad)** | Two parents born in Sweden (Reference) | 1·00 | 1·00 |
|  | Central & South America | 0·63 (0·49–0·81) | 0·71 (0·55–0·91) |
|  | Eastern Europe | 0·71 (0·58–0·87) | 0·85 (0·68–1·05) |
|  | Other Western | 0·85 (0·76–0·96) | 0·91 (0·81–1·02) |
|  | Other Nordic | 0·98 (0·71–1·35) | 1·01 (0·73–1·40) |
|  | Asia and Oceania | 1·00 (0·89–1·13) | 1·07 (0·95–1·20) |
|  | Sub-Saharan Africa | 1·01 (0·90–1·13) | 1·13 (1·01–1·27) |
|  | Finland | 0·99 (0·78–1·24) | 1·08 (0·85–1·38) |
|  | Middle East & Northern Africa | 1·23 (1·13–1·32) | 1·31 (1·22–1·42) |
| **G2.5**  **(One parent born abroad)** | Two parents born in Sweden (Reference) | 1·00 | 1·00 |
|  | Sweden | 0·80 (0·75–0·87) | 1·06 (0·98–1·14) |
|  | Central & South America | 1·01 (0·80–1·27) | 0·66 (0·50–0·85) |
|  | Eastern Europe | 1·01 (0·81–1·25) | 0·79 (0·55–1·12) |
|  | Other Western | 0·97 (0·82–1·15) | 0·90 (0·79–1·02) |
|  | Other Nordic | 1·11 (0·90–1·36) | 1·09 (0·89–1·33) |
|  | Asia and Oceania | 0·99 (0·85–1·15) | 0·85 (0·68–1·07) |
|  | Sub-Saharan Africa | 0·75 (0·49–1·14) | 0·58 (0·40–0·82) |
|  | Finland | 1·14 (0·97–1·35) | 0·84 (0·69–1·03) |
|  | Middle East & Northern Africa | 0·61 (0·40–0·93) | 0·88 (0·73–1·05) |
|  | Missing father's origin | ·· | 7·29 (6·83–7·79) |
| **Sex** | Male (Reference) | 1·00 | 1·00 |
|  | Female | 0·82 (0·79–0·85) | 0·82 (0·80–0·85) |
| **Birth order** |  | 1·00 (0·98–1·02) | 1·04 (1·03–1·06) |
| **Birth year** |  | 0·97 (0·97–0·97) | 0·96 (0·96–0·97) |
| **Maternal age** |  | 1·01 (1·01–1·02) | 1·00 (1·00–1·01) |
| **Residence** | Urban (Reference) | 1·00 | 1·00 |
|  | Rural | 1·24 (1·20–1·29) | 1·26 (1·21–1·30) |
| **Marital status** | Married or partnered (Reference) | 1·00 | 1·00 |
|  | Cohabiting | 0·47 (0·45–0·49) | 0·54 (0·51–0·56) |
|  | Single | 1·93 (1·83–2·04) | 1·72 (1·62–1·82) |
| **Mother’s education** | Low (Reference) | 1·00 | 1·00 |
|  | Medium | 0·89 (0·85–0·94) | 0·90 (0·86–0·94) |
|  | High | 0·77 (0·73–0·82) | 0·76 (0·72–0·81) |
|  | Missing | 0·86 (0·77–0·96) | 0·72 (0·66–0·80) |
| **Household disposable income quintile** | Lowest (Reference) | 1·00 | 1·00 |
|  | Lower | 0·91 (0·86–0·96) | 0·95 (0·90–1·01) |
|  | Middle | 0·82 (0·77–0·87) | 0·87 (0·81–0·93) |
|  | Higher | 0·87 (0·81–0·94) | 0·92 (0·86–0·99) |
|  | Highest | 0·94 (0·87–1·01) | 1·00 (0·93–1·07) |

*^a^* If one parent was born in Sweden, the other parent was born abroad

# **Cause of Death Descriptive Statistics**

**Table S8.** Top five underlying causes of death by age group, for children born in Sweden with two parents born in Sweden, 1990-2019.

| **Age group** | **Underlying cause of death** | **ICD-10** | **% Deaths** |
| --- | --- | --- | --- |
| Infant | Other ill-defined and unspecified causes of mortality | R99 | 6·27% |
| Infant | Sudden infant death syndrome (SIDS) | R95 | 5·11% |
| Infant | Birth asphyxia | P21 | 3·25% |
| Infant | Congenital malformations of pulmonary and tricuspid valves | Q22 | 3·19% |
| Infant | Edwards syndrome and Patau syndrome | Q91 | 2·98% |
| Ages 1-4 | Other ill-defined and unspecified causes of mortality | R99 | 4·36% |
| Ages 1-4 | Malignant neoplasm of brain | C71 | 4·18% |
| Ages 1-4 | Disorders of sphingolipid metabolism and other lipid storage disorders | E75 | 4·18% |
| Ages 1-4 | Exposure to other forces of nature | X39 | 2·14% |
| Ages 1-4 | Lymphoid leukaemia | C91 | 1·95% |
| Ages 5-14 | Malignant neoplasm of brain | C71 | 10·76% |
| Ages 5-14 | Exposure to other forces of nature | X39 | 5·06% |
| Ages 5-14 | Lymphoid leukaemia | C91 | 4·13% |
| Ages 5-14 | Epilepsy and recurrent seizures | G40 | 2·49% |
| Ages 5-14 | Malignant neoplasm of adrenal gland | C74 | 2·35% |

*Note.* Percentages indicate the share of deaths within each age group. Total deaths: infants = 6208, ages 1-4 = 1077, ages 5-14 = 1403.

**Table S9.** Top five underlying causes of death by age group, for children with both parents born abroad (G2.0), 1990-2019.

| **Age group** | **Underlying cause of death** | **ICD-10** | **% Deaths** |
| --- | --- | --- | --- |
| Infant | Other ill-defined and unspecified causes of mortality | R99 | 5·04% |
| Infant | Edwards syndrome and Patau syndrome | P02 | 3·19% |
| Infant | Foetus and newborn affected by complications of placenta, cord and membranes | Q91 | 3·19% |
| Infant | Sudden infant death syndrome (SIDS) | R95 | 2·89% |
| Infant | Birth asphyxia | P21 | 2·81% |
| Ages 1-4 | Other ill-defined and unspecified causes of mortality | R99 | 9·05% |
| Ages 1-4 | Other degenerative diseases of nervous system, not elsewhere classified | G31 | 2·88% |
| Ages 1-4 | Malignant neoplasm of brain | C71 | 2·47% |
| Ages 1-4 | Myeloid leukaemia | C92 | 2·47% |
| Ages 1-4 | Disorders of sphingolipid metabolism and other lipid storage disorders | E75 | 2·47% |
| Ages 5-14 | Malignant neoplasm of brain | C71 | 6·17% |
| Ages 5-14 | Other ill-defined and unspecified causes of mortality | R99 | 5·29% |
| Ages 5-14 | Lymphoid leukaemia | C91 | 3·52% |
| Ages 5-14 | Disorders of sphingolipid metabolism and other lipid storage disorders | E75 | 3·52% |
| Ages 5-14 | Malignant neoplasm of adrenal gland | C74 | 3·52% |

*Note.* Percentages indicate the share of deaths within each age group. Total G2.0 deaths: infants = 1350, ages 1-4 = 243, ages 5-14 = 227.

**Table S10.** Underlying causes of death by age group, for children with one parent born abroad (G2.5), 1990-2019.

| **Age group** | **Underlying cause of death** | **ICD-10** | **% Deaths** |
| --- | --- | --- | --- |
| Infant | Other ill-defined and unspecified causes of mortality | R99 | 5·85% |
| Infant | Sudden infant death syndrome (SIDS) | R95 | 4·89% |
| Infant | Foetus and newborn affected by complications of placenta, cord and membranes | P02 | 3·45% |
| Infant | Edwards syndrome and Patau syndrome | Q91 | 3·36% |
| Infant | Congenital malformations of pulmonary and tricuspid valves | Q22 | 3·26% |
| Ages 1-4 | Other ill-defined and unspecified causes of mortality | R99 | 5·95% |
| Ages 1-4 | Disorders of sphingolipid metabolism and other lipid storage disorders | E75 | 3·57% |
| Ages 1-4 | Other and unspecified infectious diseases | B99 | 2·38% |
| Ages 1-4 | Malignant neoplasm of brain | C71 | 2·38% |
| Ages 1-4 | Cerebral cysts | G93 | 1·79% |
| Ages 5-14 | Malignant neoplasm of brain | C71 | 7·62% |
| Ages 5-14 | Exposure to other and unspecified forces of nature | X39 | 4·93% |
| Ages 5-14 | Cerebral palsy | G80 | 4·04% |
| Ages 5-14 | Malignant neoplasm of adrenal gland | C74 | 3·59% |
| Ages 5-14 | Epilepsy and recurrent seizures | G40 | 3·59% |

*Note.* Percentages indicate the share of deaths within each age group. Total G2.5 deaths: infants = 1043, ages 1-4 = 168, ages 5-14 = 223.
